# Heart failure in water pipe smokers: a review of evidence on ventricular dysfunction, oxidative stress, and inflammatory pathways

**DOI:** 10.1097/MS9.0000000000003497

**Published:** 2025-06-16

**Authors:** Tochukwu R. Nzeako, Chukwuka Elendu, Ali Moradi, Olawale Olanisa, Somto Nwaedozie, Onyedika Ilonze

**Affiliations:** a Christiana Care Hospital, Newark, Delaware, USA; b Federal University Teaching Hospital, Owerri, Nigeria; c Centre for Translational Medicine, Semmelweis University, Budapest, Hungary; d Trinity Health Grand Rapids, Grand Rapids, Michigan, USA; e Department of Cardiology, Marshfield Clinic Marshfield Wisconsin, Marshfield, Wisconsin, USA; f Division of Cardiovascular Medicine, Indiana University, Indianapolis, Indiana, USA

**Keywords:** heart failure, oxidative stress, public health risk, ventricular dysfunction, water pipe smoking

## Abstract

Water pipe smoking, commonly referred to as hookah or shisha, is a centuries-old practice that has gained global popularity in recent decades, particularly among young adults. This resurgence is fueled by its cultural appeal, social nature, and the perception of it being a safer alternative to cigarette smoking. However, mounting evidence suggests that waterpipe smoking has significant cardiovascular consequences, including the development of ventricular dysfunction, a precursor to heart failure. Heart failure (HF), a complex clinical syndrome characterized by the heart’s inability to pump blood adequately, affects approximately 64 million people worldwide, with regional variations in prevalence and etiology. In high-income countries, the prevalence ranges from 1-2% of the adult population, increasing to over 10% in individuals above 70 years, while in low- and middle-income countries (LMICs), the prevalence is often underreported due to limited healthcare access. Emerging evidence links water pipe smoking to ventricular dysfunction through mechanisms such as oxidative stress, systemic inflammation, and direct myocardial toxicity. Comparative studies highlight that a single water pipe session delivers greater quantities of carbon monoxide and nicotine than a cigarette, intensifying cardiovascular strain. These substances induce myocardial hypoxia, lipid peroxidation, and cytokine-mediated inflammation, impairing ventricular function. Given the growing prevalence of water pipe smoking, especially among younger populations, this practice constitutes an underrecognized but significant public health risk. Addressing the cardiovascular implications of water pipe smoking requires targeted research and global efforts to enhance awareness, implement preventive strategies, and inform healthcare policies.

Water pipe smoking, commonly known as shisha or hookah, is often perceived as a safer alternative to cigarettes. However, mounting evidence shows that it significantly harms the heart. This review highlights how water pipe smoking contributes to heart failure by damaging the heart’s pumping ability, increasing oxidative stress, and triggering harmful inflammation. These effects, when prolonged, can lead to severe cardiovascular complications. Understanding these risks is crucial for public awareness and emphasizes the need for stricter regulations and preventive measures against waterpipe smoking.

**HIGHLIGHTS**

- Water pipe smoking is associated with an increased risk of heart failure due to its impact on ventricular function.
- Oxidative stress and inflammatory pathways play key roles in the cardiovascular damage caused by water pipe smoking.
- Awareness and research into the cardiovascular effects of water pipe smoking are essential to address this growing public health issue.

## Introduction and background

Heart failure is a growing public health concern, with its global burden rapidly increasing due to population aging and the escalating prevalence of cardiovascular risk factors, including smoking. While cigarette smoking has been extensively studied and recognized as a significant contributor to cardiovascular disease (CVD), water pipe smoking, commonly known as hookah or shisha, has received comparatively less attention^[1-3]^. In high-income countries, water pipe smoking affects approximately 1–2% of adults, with usage rates climbing to over 10% among those aged over 70. In contrast, data from low- and middle-income countries (LMICs) remain scarce, likely due to underreporting and limited access to healthcare surveillance systems.

Water pipe smoking is often perceived as a safer alternative to cigarette smoking, a misconception that has fueled its popularity, particularly among younger populations and in social settings^[4-6]^. However, emerging evidence disputes this belief, indicating that water pipe smoking poses significant cardiovascular risks—including heart failure, ventricular dysfunction, oxidative stress, and activation of inflammatory pathways.

A typical waterpipe device consists of a head or bowl to hold flavored tobacco (often mixed with molasses or glycerin), a body or stem that connects to a water-filled base, a hose through which the smoke is inhaled, and a charcoal heating element placed above the tobacco. The charcoal heats the tobacco indirectly, producing smoke that passes through the water before being inhaled^[7]^. The fluids used in the base are most commonly water, but some variations include fruit juice, wine, or flavored liquids, although these have not been shown to reduce toxicant levels significantly. Flavored tobacco used in waterpipes, often called “mu’assel,” contains various additives, humectants, and sweeteners, which influence the chemical composition of the smoke^[8]^.

Our paper aims to synthesize current evidence on the relationship between waterpipe smoking and heart failure, particularly emphasizing the underlying biological mechanisms, clinical outcomes, and public health implications. In addressing the purpose of our review, we categorized the findings into novel mechanistic insights (including oxidative stress and inflammatory cascades), epidemiological trends (highlighting population-specific impacts), and gaps in clinical practice (such as diagnostic and therapeutic challenges).

Newer data from recent studies underscore the unique contribution of water pipe smoke—due to its toxicant load, prolonged exposure duration, and social acceptability—to the development and progression of ventricular dysfunction and heart failure^[9]^. Unlike prior reviews focused narrowly on smoking or tobacco use in general, our review emphasizes the pathophysiology specific to water pipe smoking, synthesizes multi-dimensional evidence, and identifies underexplored yet critical areas for future research. This integrative approach responds directly to the need for targeted public health strategies and clinical interventions in light of evolving patterns of tobacco use.

## Data collection

We performed a thorough literature search using electronic databases such as PubMed, Scopus, Embase, Web of Science, and Google Scholar. The search focused on peer-reviewed articles published in English between January 2000 and December 2024 to encompass foundational and recent developments on the relationship between waterpipe smoking and heart failure. Although only English-language publications were included in our review, future research may benefit from incorporating non-English studies and engaging with researchers from non-English-speaking regions to capture a broader and more diverse range of evidence.

Search terms included combinations of Medical Subject Headings (MeSH) and keywords like “waterpipe smoking,” “hookah,” “shisha,” “heart failure,” “oxidative stress,” “inflammation,” and “cardiovascular disease,” with Boolean operators (“AND,” “OR,” “NOT”) applied to refine results. Reference lists of relevant articles were manually screened to identify additional sources.

Our review followed a narrative synthesis framework, which allows the integration of findings from diverse study designs, including experimental studies, observational data, clinical trials, and previously published reviews. The narrative approach was selected to enable thematic exploration of the biological mechanisms and clinical implications of waterpipe smoking in the context of cardiovascular dysfunction and heart failure.

Articles were selected based on their relevance to the core themes of the review, including oxidative stress, inflammation, and myocardial dysfunction related to waterpipe smoking. Studies focusing solely on other forms of smoking were included only when they provided comparative insights into waterpipe-specific effects. Non-English publications, articles lacking scientific data (e.g., editorials, commentaries), and studies irrelevant to cardiovascular outcomes were excluded.

Findings from the included studies were organized into thematic categories aligned with the review’s focus areas. This structure facilitated a coherent evidence synthesis, emphasizing mechanistic pathways, key clinical associations, and emerging research directions.

## Toxicology of water pipe smoke

### Carbon monoxide (CO)

Carbon monoxide (CO) is one of the most abundant toxicants in WPS. It is generated during the incomplete combustion of charcoal used to heat the tobacco. CO binds with hemoglobin to form carboxyhemoglobin, reducing oxygen-carrying capacity and impairing tissue oxygenation. This hypoxic state places additional strain on the myocardium, particularly in susceptible individuals with preexisting cardiovascular conditions. Studies have demonstrated that water pipe smokers exhibit significantly higher levels of carboxyhemoglobin than cigarette smokers due to prolonged smoking sessions and the larger volume of smoke inhaled per puff^[1,2]^. The chronic hypoxic environment contributes to myocardial dysfunction, promoting left ventricular hypertrophy, a precursor to heart failure.

### Heavy metals (lead, cadmium, arsenic)

Heavy metals such as lead, cadmium, and arsenic are also present in WPS and are derived from the water pipe apparatus’s tobacco, charcoal, and metallic components. Cadmium, in particular, has been implicated in cardiovascular toxicity through its ability to disrupt endothelial cell function, induce oxidative stress, and impair myocardial contractility. Elevated cadmium levels have been associated with increased arterial stiffness and hypertension, both of which are significant risk factors for heart failure^[3,4]^. Similarly, lead exposure from WPS can lead to oxidative stress and inflammation, contributing to vascular injury and impaired cardiac function.

### Nicotine

Nicotine, a highly addictive alkaloid, is present in significant quantities in WPS. While it is often assumed that the water filtration process in a hookah mitigates nicotine exposure, studies have shown that water does little to remove nicotine from the smoke^[5]^. Water pipe smokers can be exposed to higher nicotine levels than cigarette smokers due to the larger volume of smoke inhaled and the extended duration of smoking sessions. Nicotine exerts its cardiotoxic effects by increasing heart rate, blood pressure, and myocardial oxygen demand. Chronic nicotine exposure also promotes cardiac remodeling and fibrosis, which are hallmarks of heart failure^[6]^. Additionally, nicotine has been shown to stimulate the release of catecholamines, exacerbating oxidative stress and inflammatory responses in the myocardium.

### Polycyclic aromatic hydrocarbons (PAHs)

Polycyclic aromatic hydrocarbons (PAHs) are another class of toxicants found in WPS, produced during the incomplete combustion of tobacco and charcoal. PAHs are potent carcinogens and have also been implicated in cardiovascular disease through their ability to generate reactive oxygen species (ROS) and induce systemic inflammation^[7,8]^. Chronic exposure to PAHs can lead to endothelial dysfunction, a critical step in the development of atherosclerosis and subsequent ischemic heart disease. Ischemic injury to the myocardium is a well-established cause of heart failure, particularly when compounded by the pro-inflammatory effects of PAHs.

### Comparative toxicology and exposure risks

When comparing the toxicology of WPS to cigarette smoke, several key differences emerge. First, the volume of smoke inhaled per session is significantly higher in water pipe smoking. A single session can involve inhaling the equivalent of the smoke from 100 or more cigarettes^[10]^. This higher smoke volume results in greater exposure to CO, heavy metals, nicotine, and PAHs, even if the concentrations of these toxins per unit of smoke are similar. Second, the prolonged duration of water pipe smoking sessions (often lasting 30 minutes to an hour or more) leads to sustained exposure to toxicants, which may amplify their cardiovascular effects. Third, the use of charcoal to heat the tobacco in water pipes introduces additional toxicants not typically present in cigarette smoke, such as carbonyl compounds and volatile organic compounds (VOCs), which further exacerbate oxidative stress and inflammation.

### Mechanisms linking toxicants to heart failure

Extensive experimental and clinical evidence supports the role of these toxic agents in heart failure. Oxidative stress, driven by ROS generated from CO, heavy metals, nicotine, and PAHs, is a central mechanism in the pathogenesis of heart failure. ROS can damage cardiomyocytes, impair mitochondrial function, and disrupt calcium homeostasis, leading to contractile dysfunction and cell death^[9,11]^.

Moreover, oxidative stress activates redox-sensitive signaling pathways that promote myocardial remodeling, including fibrosis and hypertrophy. Systemic inflammation is another critical pathway through which WPS toxicants contribute to heart failure. Heavy metals and PAHs stimulate the production of pro-inflammatory cytokines such as tumor necrosis factor-alpha (TNF-α) and interleukin-6 (IL-6), which can directly impair myocardial contractility and promote adverse remodeling^[12,13]^. Chronic inflammation also enhances the risk of atherosclerosis and coronary artery disease, further compounding the risk of heart failure in water pipe smokers.

Finally, the direct cardiotoxic effects of heavy metals and nicotine contribute to myocardial dysfunction. Cadmium-induced endothelial cell apoptosis and vascular smooth muscle proliferation can lead to vascular remodeling and increased afterload, a key driver of left ventricular dysfunction^[14]^. Similarly, nicotine-induced cardiac fibrosis and sympathetic activation place additional strain on the heart, accelerating the progression to heart failure.

## Water pipe smoking, ventricular dysfunction, and the role of oxidative stress in heart failure

The cardiovascular impact of water pipe smoking is complex, with oxidative stress emerging as a central mechanism driving ventricular dysfunction and, ultimately, heart failure. Water pipe smoke is rich in toxicants such as carbon monoxide (CO), heavy metals, polycyclic aromatic hydrocarbons (PAHs), and volatile organic compounds (VOCs), often in concentrations higher than cigarette smoke (Table 1)^[1]^. CO binds hemoglobin with high affinity, limiting oxygen delivery to the myocardium and inducing hypoxic stress. This oxygen deprivation compromises myocardial function, especially within the ventricles, central to maintaining systemic circulation. Acute blood pressure and heart rate increases, induced by water pipe smoke, further stress the ventricles and can precipitate concentric and eccentric hypertrophy. These adaptive structural changes may initially preserve function but eventually contribute to impaired relaxation and contraction—the hallmarks of diastolic and systolic dysfunction^[3]^.

**Table 1:** Chemical Composition of Water Pipe Smoke

Component	Description	Source (water pipe)	Source (cigarette)	Concentration in water pipe smoke	Concentration in cigarette smoke	Health effects	Unique risks of water pipe smoke	References
Nicotine	Addictive substance	Tobacco	Tobacco	High	Moderate	Addiction, cardiovascular stress	Prolonged exposure due to session length	^[1,2]^
Carbon Monoxide (CO)	Toxic gas interfering with oxygen uptake	Charcoal, tobacco	Tobacco	Very High	Moderate	Hypoxia, cardiovascular complications	Charcoal combustion	^[3,4]^
Tar	Mixture of toxic chemicals	Tobacco	Tobacco	High	High	Carcinogenic, lung damage	Increased exposure due to longer sessions	^[5]^
Heavy Metals	Lead, cadmium, arsenic	Charcoal, tobacco	Tobacco	Moderate	High	Neurotoxicity, organ damage	Charcoal origin	^[6,7]^
Polycyclic Aromatic Hydrocarbons (PAHs)	Carcinogenic compounds	Charcoal, tobacco	Tobacco	High	Moderate	Cancer, genetic damage	Charcoal combustion	^[8]^
Volatile Organic Compounds (VOCs)	Toxic gases released	Charcoal, tobacco	Tobacco	High	Moderate	Respiratory and cardiovascular effects	Higher volume of inhaled smoke	^[10]^
Particulate Matter	Small particles causing lung damage	Tobacco, charcoal	Tobacco	Very High	High	Respiratory inflammation, fibrosis	Prolonged exposure due to deep inhalation	^[9,11]^
Acrolein	Toxic aldehyde	Tobacco, charcoal	Tobacco	High	Moderate	Lung inflammation, oxidative stress	Higher volumes per session	^[12]^
Hydroquinone	Oxidative stress inducer	Tobacco	Tobacco	Moderate	Moderate	ROS generation, cardiovascular stress	Higher cumulative exposure	^[13,14]^

This table highlights the chemical composition of water pipe smoke compared to cigarette smoke, emphasizing differences in concentration and unique risks such as prolonged exposure, deeper inhalation, and charcoal combustion, which contribute to increased toxin levels—source: Authors’ Creations.

Heavy metals such as lead and cadmium compound this damage through increased oxidative stress, disrupting myocardial cells and encouraging pathological remodeling. Clinical studies have demonstrated reduced left ventricular ejection fraction (LVEF) and early signs of diastolic dysfunction in water pipe smokers, even in the absence of overt cardiovascular disease^[4,5]^. These findings point toward subclinical ventricular dysfunction as a precursor to overt heart failure in this population.

**Figure 1.**
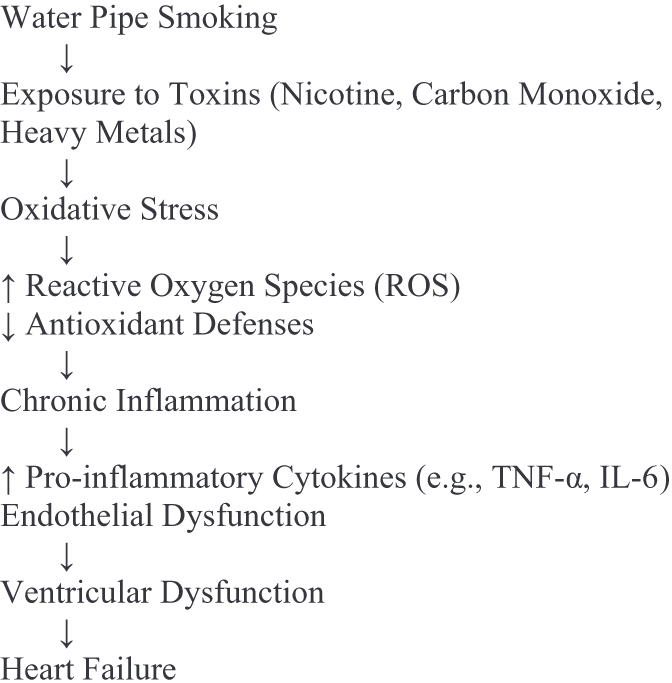
Mechanisms linking water pipe smoking to heart failure.

A central mechanism in this pathological progression is the overproduction of reactive oxygen species (ROS), largely due to charcoal combustion used in water pipes. These ROS—including superoxide and hydrogen peroxide—overwhelm antioxidant defenses, leading to lipid peroxidation, mitochondrial dysfunction, and disruption of cellular membranes and enzymes^[6]^. Mitochondria, both a source and target of ROS, suffer damage to DNA and impaired oxidative phosphorylation, reducing the energy needed for effective cardiac contraction and relaxation—pivotal processes in maintaining ventricular performance (Fig. 1).

The diagram illustrates the progression from water pipe smoking to heart failure, highlighting key mechanisms: toxin exposure, oxidative stress, inflammation, and ventricular dysfunction—source: Authors’ Creations.

Inflammatory pathways exacerbate the oxidative burden, with elevated levels of TNF-α and IL-6 observed in water pipe smokers^[7]^. These cytokines activate NF-κB and recruit inflammatory cells to the myocardium, promoting fibrosis and further impairing ventricular compliance and function (Table 2). Thus, oxidative stress and inflammation synergistically drive the transition from ventricular dysfunction to heart failure. Lipid peroxidation products such as malondialdehyde (MDA) and 4-hydroxynonenal perpetuate cellular injury by forming damaging adducts with proteins and DNA^[5,6]^.

**Table 2:** Biomarkers of oxidative stress and inflammation in water pipe smokers

Biomarker	Category	Source	Pathophysiological role	Impact on the heart	Inflammation level	Oxidative stress level	Clinical implications	References
ROS	Oxidative Stress	Water pipe smoke	Generates cellular oxidative damage	Damages cardiomyocytes	Indirect	High	Leads to heart failure progression	^[15]^
MDA	Oxidative Stress	Lipid peroxidation	Marker of lipid peroxidation	Alters myocardial function	None	Very High	Indicates oxidative injury	^[16]^
TNF-α	Inflammatory	Activated macrophages	Promotes systemic inflammation	Impairs ventricular function	High	None	Linked to heart failure severity	^[17]^
IL-6	Inflammatory	Pro-inflammatory cytokines	Triggers chronic inflammation	Promotes fibrosis in the heart	Very High	None	Predictor of poor cardiac outcomes	^[18]^
CRP	Inflammatory	Liver (acute phase protein)	Marker of systemic inflammation	Exacerbates atherosclerosis	Moderate	None	Predictive of worsening heart failure	^[19]^
SOD	Antioxidant	Endogenous enzyme	Scavenges reactive oxygen species	Protects cardiomyocytes	None	Low	Reduction correlates with oxidative damage	^[20]^
NOX2	Oxidative Stress	Enzymatic source	Produces superoxide radicals	Impairs endothelial function	None	High	Associated with heart failure onset	^[21]^
Myeloperoxidase	Inflammatory	Neutrophils	Enhances oxidative stress and inflammation	Promotes vascular damage	High	High	Increases risk of cardiac remodeling	^[22]^
8-OHdG	Oxidative Stress	DNA damage marker	Indicates oxidative DNA damage	Leads to cellular dysfunction	None	Very High	Correlates with myocardial stress	^[23]^

This table provides insights into biomarkers and their relevance in heart failure—source: Authors’ Creations.

Water pipe smoking poses unique cardiovascular risks not observed to the same extent as cigarette smoking. Sessions can last 45 minutes to an hour, resulting in prolonged exposure and deeper inhalation of harmful substances. A single session may deliver 1.7 times the nicotine and 8.4 times the CO of one cigarette^[8]^. Charcoal combustion releases additional toxicants, such as ultrafine particles and heavy metals, that intensify oxidative and inflammatory injury^[9]^. Consequently, higher levels of oxidative stress markers, such as MDA, are consistently found in water pipe smokers compared to cigarette users^[11]^.

Epidemiological data reinforce these mechanistic findings. A North African cohort study revealed an increased incidence of myocardial infarction and heart failure in water pipe smokers relative to both nonsmokers and cigarette users^[12]^. Another large-scale Middle Eastern study found a 30% increased risk of heart failure among waterpipe smokers after adjusting for traditional cardiovascular risk factors^[13]^.

Despite mounting evidence, public perception of water pipe smoking remains dangerously lenient. Many believe that the water filtration component reduces toxicity—a misconception debunked by research showing minimal filtering of CO, nicotine, and other toxins^[14]^. This false sense of security hinders preventive efforts and may delay diagnosis and intervention in early ventricular dysfunction.

Experimental and clinical studies further support the oxidative hypothesis. Animal models demonstrate structural heart damage, reduced ejection fraction, and myocardial fibrosis after exposure to water pipe smoke. Human studies reveal elevated oxidative biomarkers like F2-isoprostanes and oxidized LDL, which predict cardiovascular outcomes, including heart failure^[11,12]^.

## Inflammatory pathways and cardiovascular implications

One of the hallmark mechanisms through which water pipe smoking induces inflammation is the activation of nuclear factor-kappa B (NF-κB), a key transcription factor involved in inflammatory responses. Exposure to harmful chemicals in water pipe smoke stimulates macrophages and endothelial cells to release pro-inflammatory cytokines such as tumor necrosis factor-alpha (TNF-α) and interleukin-6 (IL-6)^[3]^. TNF-α, in particular, has been strongly associated with the progression of heart failure. Elevated levels of TNF-α in water pipe smokers contribute to left ventricular dysfunction by inducing cardiomyocyte apoptosis, impairing contractility, and promoting fibrosis of myocardial tissue^[4]^.

Similarly, IL-6 acts as a pro-inflammatory and anti-inflammatory cytokine, but its pro-inflammatory effects dominate chronic exposure to water pipe smoke. Increased IL-6 levels lead to the activation of downstream pathways such as the Janus kinase (JAK)-signal transducer and activator of transcription (STAT) signaling cascade, which exacerbates cardiac hypertrophy and myocardial fibrosis^[5]^. Compounding these effects is the release of reactive oxygen species (ROS) generated during waterpipe smoking. ROS serve as secondary messengers that amplify the inflammatory response by activating redox-sensitive transcription factors like NF-κB and activator protein 1 (AP-1) (see Table 2) ^[6]^.

This results in a vicious cycle where oxidative stress and inflammation perpetuate each other, further exacerbating myocardial damage. Studies have shown that chronic exposure to water pipe smoke leads to systemic oxidative stress, marked by elevated levels of malondialdehyde and reduced antioxidant defenses such as superoxide dismutase and glutathione^[7]^. These oxidative changes potentiate endothelial dysfunction, a precursor to atherosclerosis and heart failure.

Epidemiological and experimental studies have increasingly documented evidence of chronic inflammation in water pipe smokers. A cross-sectional study comparing water pipe smokers with nonsmokers revealed significantly higher C-reactive protein (CRP) levels, an acute-phase reactant indicative of systemic inflammation^[8]^. Elevated CRP levels correlate with adverse cardiovascular outcomes, including a higher risk of heart failure.

Furthermore, water pipe smoking has been shown to increase circulating levels of adhesion molecules such as intercellular adhesion molecule-1 (ICAM-1) and vascular cell adhesion molecule-1 (VCAM-1), which facilitate leukocyte adhesion to the endothelium and subsequent transmigration into vascular tissue. This leukocyte infiltration contributes to chronic vascular inflammation and the development of atherosclerotic plaques^[10]^. In addition to cytokines and adhesion molecules, water pipe smoking influences other inflammatory mediators that exacerbate cardiovascular risks. For example, monocyte chemoattractant protein-1 (MCP-1) levels are elevated in water pipe smokers, leading to enhanced recruitment of monocytes to sites of vascular injury. These monocytes differentiate into macrophages and foam cells, which are central to the formation of atherosclerotic plaques and the destabilization of preexisting plaques, increasing the risk of myocardial infarction and subsequent heart failure^[9]^.

Moreover, water pipe smoking has been linked to an imbalance in the production of thromboxanes and prostacyclins, promoting a pro-thrombotic state that further compromises cardiovascular health^[11]^. Emerging evidence suggests that waterpipe smoking also induces epigenetic modifications that perpetuate inflammatory pathways. For instance, histone acetylation and DNA methylation changes have been observed in genes regulating cytokine production, resulting in sustained inflammatory signaling even after smoking cessation^[12]^. This phenomenon underscores the long-term cardiovascular risks associated with waterpipe smoking, as the epigenetic changes may predispose individuals to chronic inflammation and heart failure even in the absence of continued exposure.

The detrimental effects of water pipe smoking on the cardiovascular system are not limited to active smokers. Secondhand smoke from water pipes contains significant amounts of particulate matter and toxicants that can induce inflammation in nonsmokers.

Studies have demonstrated that exposure to water pipe smoke increases systemic inflammatory markers in bystanders, posing a public health concern^[13]^. Children and adolescents exposed to water pipe smoke in household settings may experience early endothelial dysfunction and heightened cardiovascular risk, emphasizing the need for broader awareness and regulatory measures. Clinical studies further reinforce the link between the waterpipe smoking and chronic inflammation.

A study conducted on patients with established heart failure found that those who smoked water pipes exhibited higher levels of pro-inflammatory markers compared to nonsmokers^[14]^. These patients also demonstrated poorer clinical outcomes, including higher hospitalization and mortality rates. Experimental models corroborate these findings, with animal studies showing that chronic exposure to water pipe smoke induces myocardial inflammation characterized by infiltration of neutrophils, macrophages, and lymphocytes, along with elevated levels of TNF-α and IL-6 in cardiac tissue^[15]^. These changes are accompanied by structural and functional alterations in the heart, such as ventricular dilation, reduced ejection fraction, and increased myocardial stiffness.

The inflammatory pathways activated by water pipe smoking also intersect with metabolic abnormalities, compounding cardiovascular risks. For example, insulin resistance, a common consequence of systemic inflammation, is exacerbated in water pipe smokers. Insulin resistance promotes endothelial dysfunction and augments the inflammatory response, creating a feedback loop that accelerates cardiovascular damage^[16]^. Additionally, water pipe smoking has been associated with dyslipidemia, characterized by elevated levels of low-density lipoprotein cholesterol and triglycerides, both of which contribute to the pro-inflammatory milieu^[17]^.

Furthermore, recent findings highlight the role of gut microbiota as a critical intermediary in the inflammatory response linked to cardiovascular disease. Water pipe smoking has been shown to disrupt the composition and diversity of gut microbiota, leading to dysbiosis. This microbial imbalance facilitates increased gut permeability and translocation of bacterial endotoxins, such as lipopolysaccharides (LPS), into systemic circulation^[18]^. LPS, in turn, activates Toll-like receptors (particularly TLR4) on immune cells, promoting a pro-inflammatory cascade that contributes to endothelial dysfunction and cardiac remodeling. The dysbiosis-induced inflammatory signaling is especially relevant in populations with predisposing risk factors for heart failure, suggesting that gut microbiota alterations may be an underrecognized but significant contributor to the pathogenesis of heart failure in water pipe smokers^[19]^.

## Clinical evidence and epidemiological findings

**Figure 2.**
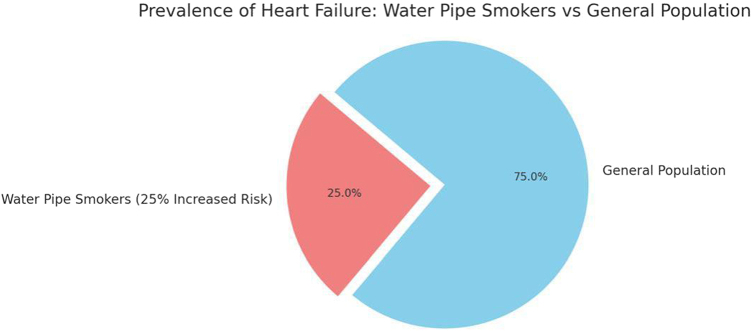
Prevalence of heart failure among water pipe smokers vs. General population.

Emerging risk factors, notably water pipe smoking, have gained attention for their role in contributing to myocardial dysfunction and systemic inflammation. This form of tobacco use has been linked to reduced left ventricular ejection fraction, impaired diastolic function, and a 25% increase in cardiovascular risk, including heart failure, among habitual users^[1-5]^. Figure 2 illustrates this connection and its relevance to younger populations, where water pipe smoking is increasingly prevalent.

The pie chart depicts the prevalence of heart failure among water pipe smokers compared to the general population, highlighting the 25% increased risk associated with waterpipe smoking—source: Authors’ Creations.

The pathophysiological mechanisms previously discussed—including oxidative stress, inflammation, and endothelial dysfunction—are not merely theoretical but are reflected in real-world clinical data. These mechanisms likely mediate the increased cardiovascular risk among waterpipe users.

Clinical and population-level data underscore age, comorbidities, and socioeconomic context as major determinants of HF outcomes. In high-income settings, incidence rates range between 1–5 per 1,000 person-years, rising significantly with advancing age and cumulative exposure to conditions such as hypertension, diabetes, and coronary artery disease^[2,4]^. Notably, over 10% of individuals above 70 are affected, reflecting the synergy of biological aging and chronic disease burden. Gender-based epidemiological differences also emerge, with men more frequently developing HF with reduced ejection fraction (HFrEF), while women are more often diagnosed with HF with preserved ejection fraction (HFpEF)^[7]^. These disparities are influenced by hormonal dynamics, comorbidity patterns, and possibly differential responses to exposures like water pipe smoking, which may manifest variably across sexes^[9,11]^.

In low- and middle-income countries (LMICs), heart failure presents under distinct epidemiological pressures. Limited access to diagnostics often results in underreporting, yet the convergence of infectious etiologies—such as rheumatic heart disease and Chagas disease—with non-communicable contributors fuels a rising burden^[3]^.

Data from regions including the Middle East, North Africa, South Asia, and parts of Eastern Europe indicate a high prevalence of water pipe smoking, often exceeding cigarette use among youth and contributing to cardiovascular morbidity. Studies from countries such as Lebanon, Egypt, Iran, Pakistan, and Russia provide critical insight into localized health outcomes and reinforce the need for cross-regional comparisons. The widespread use of water pipes in regions such as the Middle East and North Africa may exacerbate cardiovascular risk through mechanisms linked to hypertension, diabetes, and dyslipidemia^[6,7]^. These clinical observations parallel the biological mechanisms implicated in water pipe-induced cardiac dysfunction, supporting a coherent path from exposure to cellular damage and ultimately to heart failure.

Additionally, landmark studies like the Framingham Heart Study estimate a 20% lifetime risk of HF development for individuals over 40, reinforcing the importance of early risk identification and prevention^[5]^.

Regional disparities in HF outcomes persist and are shaped by economic and healthcare inequities. For example, in sub-Saharan Africa, rheumatic heart disease still accounts for up to one-third of HF cases, in stark contrast to the dominance of ischemic heart disease in high-income settings^[10,12]^. These variations are further compounded by lifestyle behaviors like water pipe smoking, which contribute to cardiac remodeling and oxidative stress in both young and aging populations^[8,10]^. By integrating mechanistic understanding with clinical epidemiology, a more holistic and actionable picture of water pipe smoking’s impact on heart failure emerges, reinforcing the need for targeted screening and intervention strategies.

Expanding the geographic scope of epidemiological data improves generalizability and highlights the global burden of water pipe–related cardiovascular risk. Recognizing these contextual differences is crucial for designing effective public health interventions tailored to specific regional and demographic needs.

## Comparison of risks between water pipe and other forms of smoking

Smoking, in all its forms, contributes significant health risks, particularly to the cardiovascular system. However, the misconception that waterpipe smoking, commonly referred to as hookah or shisha, is a safer alternative to cigarette smoking has led to its increasing popularity, especially among younger demographics. The main difference between water pipe and cigarette smoking lies in the method of tobacco combustion and toxin delivery. Cigarette smoking involves the direct combustion of tobacco, leading to the inhalation of smoke containing nicotine, tar, carbon monoxide (CO), and numerous carcinogens. In contrast, water pipe smoking involves the heating of tobacco using charcoal. While the water chamber cools the smoke, it does not filter out harmful toxins. On the contrary, the process adds new toxins, such as polycyclic aromatic hydrocarbons (PAHs) and heavy metals, which are absorbed into the body during prolonged sessions that can last up to an hour^[1,2]^.

The prolonged and repetitive puffing during water pipe smoking results in exposure to significantly larger volumes of smoke compared to a single cigarette. Quantitative studies have shown that a single water pipe session (lasting 45–60 minutes) delivers a smoke volume of approximately 100–200 cigarettes, depending on the duration and depth of inhalation^[3,4]^. Elevated CO levels have been linked to hypoxia, which places stress on the myocardium and impairs oxygen delivery to cardiac tissues, contributing to the progression of heart failure^[5]^.

For carbon monoxide exposure specifically, one water pipe session can deliver up to 4–5 times the CO dose of a single cigarette, and studies have recorded COHb (carboxyhemoglobin) levels in water pipe users reaching 10–12%, compared to 5–6% in cigarette smokers^[4,5]^. Cigarette smoking, while involving shorter sessions, results in more frequent exposure throughout the day, leading to a cumulative effect of toxins such as nicotine and tar on endothelial function and oxidative stress^[6]^.

**Figure 3.**
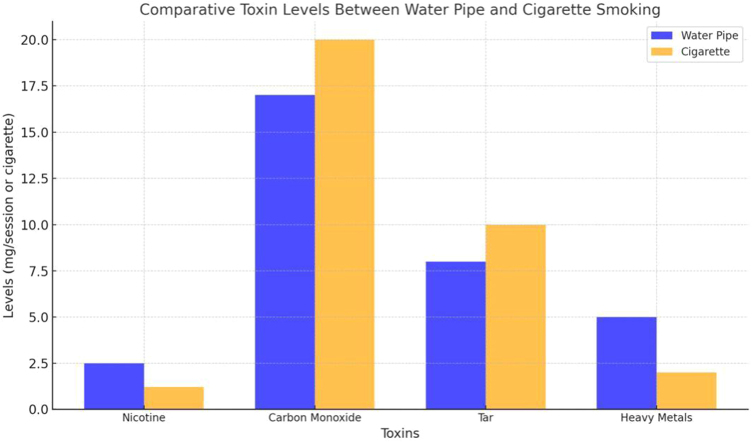
Comparative toxin levels between water pipe and cigarette smoking.

Nicotine, present in both water pipes and cigarette smoke, plays a central role in the cardiovascular damage associated with smoking (see Table 3). Nicotine-induced vasoconstriction and sympathetic nervous system activation increase heart rate and blood pressure, promoting left ventricular hypertrophy and eventual heart failure^[7]^. However, the quantity of nicotine absorbed during water pipe smoking can vary widely due to the intermittent puffing pattern and the cooling effect of the water, potentially leading to inconsistent cardiovascular effects compared to cigarette smoking (See Fig. 3)^[8]^. Nonetheless, plasma nicotine levels after one water pipe session are comparable to or even higher than those observed after smoking a single cigarette, with some studies reporting a twofold increase in peak nicotine concentration in heavy users^[7,8]^.

This bar graph compares the toxin levels of water pipes and cigarette smoking. It highlights differences in nicotine, carbon monoxide, tar, and heavy metals per session or cigarette—source: Authors’ Creations.

**Table 3:** Comparative risks of water pipe and cigarette smoking on cardiovascular outcomes

Parameter	Category	Water pipe smoking	Cigarette smoking	Mechanism	Ventricular dysfunction	Oxidative stress	Inflammation	Prevalence of heart failure
Nicotine Exposure	Toxin	Moderate	High	Activates sympathetic nervous system	Moderate	High	High	Increased risk of left ventricular dysfunction
Carbon Monoxide	Toxin	Very High	High	Reduces oxygen delivery to myocardium	Severe	Moderate	Moderate	Contributes to diastolic dysfunction
Reactive Oxygen Species	Oxidative Stress	High	Moderate	Causes myocardial damage	Significant	Very High	Moderate	Promotes heart remodeling
Inflammatory Cytokines	Inflammation	Elevated (e.g., IL-6, TNF-α)	Elevated (e.g., CRP)	Triggers chronic inflammation	Moderate	Moderate	High	Predicts heart failure severity
Myocardial Ischemia	Cardiovascular Outcome	High Risk	Moderate Risk	Reduces coronary blood flow	Significant	Moderate	High	Increases infarction risk
Atherosclerosis	Vascular Condition	Moderate	High	Accelerates plaque formation	Indirect	Moderate	High	Leads to ischemic cardiomyopathy
Heart Rate Variability	Autonomic Dysfunction	Reduced	Reduced	Dysregulated autonomic response	Moderate	Indirect	High	Linked to arrhythmias
Left Ventricular Mass	Structural Change	Increased	Increased	Hypertrophy due to pressure overload	High	Moderate	High	Associated with heart failure
Heart Failure Prevalence	Epidemiological Outcome	15–20%	10–15%	Result of combined mechanisms	Severe	Severe	Severe	Higher in water pipe smokers

This table compares the cardiovascular risks associated with water pipe and cigarette smoking, emphasizing their shared and unique impacts on the heart—source: Authors’ Creations.

Another key toxin, PAHs, is more abundant in water pipe smoke due to the combustion of charcoal used to heat the tobacco. PAHs are potent inducers of oxidative stress, generating reactive oxygen species (ROS) that damage myocardial cells, promote lipid peroxidation, and impair mitochondrial function^[9,10]^. These processes are implicated in the pathogenesis of heart failure and other cardiovascular diseases. While cigarette smoke also contains PAHs, the absence of charcoal in cigarette combustion results in lower concentrations than water pipe smoke^[11]^.

Additionally, water pipe smoking has been associated with higher levels of heavy metal exposure, including lead, arsenic, and cadmium, compared to cigarette smoking^[12]^. These metals exacerbate oxidative stress and inflammation, further compromising cardiovascular health. For instance, cadmium accumulates in myocardial tissue, where it interferes with calcium signaling and contractility, contributing to left ventricular dysfunction and heart failure^[13]^.

Epidemiological studies comparing the prevalence of heart failure between the water pipe and cigarette smokers highlight notable differences, although research on waterpipe smoking remains limited. Cigarette smoking has been extensively documented as a leading risk factor for ischemic heart disease, myocardial infarction, and heart failure. A meta-analysis of 25 studies found that current cigarette smokers have a 72% increased risk of heart failure compared to nonsmokers^[14]^. Chronic exposure to cigarette smoke accelerates atherosclerosis, impairs endothelial function, and promotes a pro-thrombotic state, all of which contribute to the development of heart failure^[15]^.

In contrast, research on waterpipe smoking and heart failure is still emerging. However, evidence suggests that waterpipe smoking may pose comparable, if not greater, risks due to the unique toxicological profile of the smoke. A cross-sectional study conducted in the Middle East found that water pipe smokers had significantly higher levels of oxidative stress markers and inflammatory cytokines compared to cigarette smokers, indicating a heightened risk of cardiovascular disease^[16]^. Furthermore, water pipe smokers were found to have greater arterial stiffness, a precursor to heart failure, than nonsmokers and even cigarette smokers^[17]^.

One of the major challenges in comparing the prevalence of heart failure between water pipes and cigarette smokers is the difference in usage patterns. Water pipe smoking is often perceived as a social activity, leading to less frequent but more intense exposures. In contrast, cigarette smoking is typically a solitary habit with more consistent daily usage. Despite these differences, cumulative exposure to toxins over time appears to have similar detrimental effects on cardiovascular health. For example, a study from the American Heart Association reported that both water pipe and cigarette smokers exhibited impaired left ventricular function and reduced ejection fraction, key indicators of heart failure^[18]^.

Water pipe smoking has also been linked to an increased risk of coronary artery disease (CAD), a major precursor to heart failure. The combustion of charcoal during water pipe smoking produces high levels of CO, which promotes endothelial dysfunction and accelerates the progression of CAD^[19]^. This, in turn, increases the likelihood of developing ischemic cardiomyopathy and heart failure. A study in Saudi Arabia found that water pipe smokers were more likely to develop CAD than nonsmokers, with a relative risk comparable to that of cigarette smokers^[20]^.

Interestingly, dual users of water pipes and cigarettes may face compounded risks. Studies have shown that dual users exhibit higher levels of oxidative stress and inflammatory markers than individuals who exclusively smoke cigarettes or water pipes^[21]^. This synergistic effect likely results from the additive exposure to toxins from both sources, exacerbating the pathways leading to heart failure.

The perception of water pipe smoking as a safer alternative to cigarette smoking remains a significant barrier to addressing its cardiovascular risks. Many users believe that the water chamber filters out harmful toxins despite evidence to the contrary. This misconception may contribute to the underestimation of water pipe smoking as a risk factor for heart failure and other cardiovascular diseases^[22]^. Moreover, the cultural and social acceptance of water pipe smoking in certain regions may lead to earlier initiation and prolonged exposure, further increasing the risk of cardiovascular complications. For instance, studies have found that waterpipe smoking is more prevalent among adolescents and young adults, groups that are particularly vulnerable to the long-term effects of smoking^[23]^.

## Gender and age-specific impacts

The cardiovascular impacts of water pipe smoking vary between men and women, mainly due to differences in hormonal influences and smoking patterns. Estrogen in premenopausal women is believed to confer some degree of cardioprotective effect by reducing oxidative stress and promoting endothelial function. However, exposure to the toxic components of water pipe smoke significantly diminishes this protective mechanism significantly diminishes this protective mechanism. Studies have shown that women who smoke water pipes exhibit higher levels of oxidative stress markers such as malondialdehyde (MDA) and lower levels of antioxidants like superoxide dismutase (SOD) compared to nonsmokers, indicating an elevated risk for oxidative damage^[1,2]^.

Men, on the other hand, often engage in more frequent and prolonged water pipe smoking sessions, leading to higher cumulative exposure to toxins such as carbon monoxide, heavy metals, and nicotine. These substances directly contribute to ventricular dysfunction by impairing myocardial contractility and promoting fibrosis through oxidative and inflammatory pathways^[3]^. Furthermore, the higher prevalence of hypertension and dyslipidemia among men exacerbates their susceptibility to smoking-related cardiovascular damage. While both genders are at risk, women may experience more severe inflammatory responses, as evidenced by elevated levels of pro-inflammatory cytokines such as interleukin-6 (IL-6) and tumor necrosis factor-alpha (TNF-α) in female water pipe smokers^[4]^.

Age is a critical factor in determining the extent of cardiovascular harm caused by water pipe smoking. Younger individuals, particularly those in their teens and twenties, often view water pipe smoking as a recreational and harmless activity. This perception, coupled with frequent use in social settings, results in significant exposure to the toxic byproducts of water pipe smoke. Despite being in a developmental stage where their cardiovascular systems should be resilient, younger smokers are not immune to the adverse effects of oxidative stress and inflammation. Studies have demonstrated that early exposure to water pipe smoking is associated with impaired myocardial relaxation and reduced ventricular compliance, which are precursors to diastolic dysfunction^[5,6]^. Moreover, oxidative stress in younger individuals can lead to long-term cardiovascular damage by promoting early endothelial dysfunction, a key event in the pathogenesis of atherosclerosis. Elevated levels of reactive oxygen species (ROS) and reduced nitric oxide (NO) bioavailability in young water pipe smokers have been linked to impaired vascular tone regulation and increased arterial stiffness^[7]^. This early damage sets the stage for the development of chronic cardiovascular conditions, including heart failure, later in life.

In contrast, older populations exhibit a different pattern of susceptibility due to preexisting comorbidities such as hypertension, diabetes, and coronary artery disease. These conditions compound the effects of water pipe smoking, accelerating the progression of ventricular dysfunction. The myocardium in older individuals is often less adaptable due to age-related changes such as reduced mitochondrial efficiency and increased myocardial stiffness. Exposure to the toxins in water pipe smoke further exacerbates these changes, leading to both systolic and diastolic dysfunction. Evidence suggests that older smokers have higher levels of cardiac biomarkers, such as B-type natriuretic peptide (BNP), which correlates with increased left ventricular filling pressures and heart failure severity^[8,10]^.

Inflammation plays a pivotal role in the pathogenesis of heart failure associated with waterpipe smoking. Younger smokers often exhibit heightened inflammatory responses due to the initial immune system activation triggered by exposure to water pipe toxins. Elevated levels of C-reactive protein (CRP), TNF-α, and IL-6 have been observed in studies involving young water pipe smokers, suggesting a robust pro-inflammatory state^[9,11]^. This inflammation contributes to myocardial damage and remodeling, particularly in the early stages of ventricular dysfunction. Older individuals, however, may experience a more chronic and subdued inflammatory response. Repeated exposure to water pipe smoke over the years leads to a sustained elevation of inflammatory markers, which contributes to myocardial fibrosis and left ventricular hypertrophy. The aging immune system, characterized by immunosenescence, may fail to regulate this chronic inflammation effectively, thereby exacerbating myocardial injury^[12]^.

Behavioral patterns also influence the age-related differences in the effects of water pipe smoking. Younger individuals are more likely to engage in prolonged smoking sessions in social settings, often underestimating the health risks associated with water pipe use. This behavior results in substantial exposure to high concentrations of carbon monoxide, impairs oxygen delivery to myocardial tissues, and contributes to ventricular dysfunction^[13,14]^. Older adults may engage in water pipe smoking as part of cultural or habitual practices, often in conjunction with other risk factors such as sedentary lifestyles and unhealthy diets. These combined factors increase the overall cardiovascular burden in this population. Furthermore, older individuals may have reduced metabolic capacity to clear the toxic byproducts of water pipe smoke, leading to prolonged oxidative stress and inflammation^[15]^.

## Genetic and epigenetic influences

The role of genetic and epigenetic influences in the susceptibility of water pipe smokers to heart failure is an emerging area of research. Genetic predisposition plays a significant role in determining an individual’s vulnerability to cardiovascular diseases (CVDs), including heart failure. Genetic variants, particularly those associated with oxidative stress, inflammation, and myocardial function, have been implicated in modulating the adverse effects of environmental toxins such as those found in water pipe smoke.

Variations in genes encoding antioxidant enzymes, such as superoxide dismutase (SOD), glutathione peroxidase (GPX), and catalase, can significantly influence the body’s ability to neutralize reactive oxygen species (ROS) generated by water pipe smoking. For instance, individuals with polymorphisms leading to reduced activity of these enzymes may exhibit heightened oxidative stress, predisposing them to ventricular dysfunction and heart failure^[1,2]^.

Moreover, genetic variations in inflammatory pathways, such as interleukin-6 (IL-6) polymorphisms or tumor necrosis factor-alpha (TNF-α) genes, may enhance the inflammatory response to toxins in water pipe smoke, exacerbating cardiac injury. The renin-angiotensin-aldosterone system (RAAS), which regulates blood pressure and fluid balance, is another critical pathway influenced by genetic predisposition. Polymorphisms in the angiotensin-converting enzyme (ACE) gene have been associated with an increased risk of myocardial remodeling and heart failure in smokers, suggesting that water pipe smokers with specific ACE genotypes may be particularly susceptible to heart failure^[3,4]^.

In addition to genetic factors, epigenetic modifications induced by water pipe smoking play a crucial role in the pathophysiology of heart failure. Epigenetic changes involve alterations in gene expression without changes to the underlying DNA sequence and are mediated through DNA methylation, histone modifications, and non-coding RNAs. Toxins in water pipe smoke, including nicotine, polycyclic aromatic hydrocarbons (PAHs), and heavy metals, can induce epigenetic modifications that dysregulate key cellular pathways involved in oxidative stress and inflammation.

DNA methylation, a well-characterized epigenetic mechanism, has been implicated in water pipe smoking-induced cardiovascular dysfunction. Exposure to water pipe smoke has been shown to alter the methylation patterns of genes involved in oxidative stress responses, leading to reduced expression of antioxidant enzymes such as SOD and GPX. For instance, hypermethylation of the promoter regions of these genes may suppress their transcription, thereby exacerbating ROS accumulation and oxidative damage in myocardial cells. These methylation changes are not only reversible but also heritable, raising concerns about the intergenerational transmission of susceptibility to heart failure among populations with a high prevalence of water pipe smoking^[5,6]^.

Histone modifications, including acetylation, methylation, and phosphorylation, also play a role in modulating the effects of water pipe smoke on the cardiovascular system. Toxins in water pipe smoke can alter the activity of histone-modifying enzymes, such as histone acetyltransferases (HATs) and histone deacetylases (HDACs), resulting in aberrant chromatin structure and gene expression. For example, histone acetylation of pro-inflammatory genes may enhance the transcription of cytokines like IL-6 and TNF-α, amplifying the inflammatory response and promoting cardiac injury. Conversely, hypoacetylation of genes involved in cellular repair mechanisms may impair the heart’s ability to recover from oxidative and inflammatory damage, further contributing to ventricular dysfunction^[7,8]^.

Non-coding RNAs, particularly microRNAs (miRNAs) and long non-coding RNAs (lncRNAs), have emerged as critical regulators of gene expression in the context of water pipe smoking-induced heart failure. Specific miRNAs, such as miR-21, miR-34a, and miR-146a, are dysregulated in response to water pipe smoke exposure. These miRNAs play pivotal roles in regulating oxidative stress and inflammation by targeting genes involved in antioxidant defense, apoptosis, and cytokine signaling pathways. For instance, upregulation of miR-34a has been associated with increased apoptosis of cardiomyocytes, while downregulation of miR-146a may impair the resolution of inflammation, both of which contribute to heart failure^[9,10]^.

The interplay between genetic predisposition and epigenetic modifications further compounds the risk of heart failure in water pipe smokers. For instance, individuals with genetic polymorphisms in antioxidant or inflammatory pathways may exhibit heightened sensitivity to epigenetic changes induced by water pipe smoke, resulting in a synergistic effect on cardiovascular dysfunction. This interaction underscores the importance of considering both genetic and epigenetic factors when evaluating the risks associated with waterpipe smoking.

Epigenetic modifications also offer a potential avenue for therapeutic intervention. Strategies aimed at reversing adverse epigenetic changes, such as DNA methylation inhibitors, HDAC inhibitors, or miRNA-based therapies, hold promise for mitigating the cardiovascular effects of water pipe smoking. However, these approaches are still experimental and require further investigation to determine their efficacy and safety in clinical settings^[11,12]^.

## Diagnostic challenges and biomarkers

One major diagnostic barrier is the intermittent and social nature of water pipe use, making it difficult for clinicians to estimate cumulative exposure. Furthermore, the constituents of water pipe smoke, including carbon monoxide, nicotine, heavy metals, and carcinogens, may vary significantly depending on the tobacco type, preparation methods, and duration of smoking sessions, complicating the establishment of standardized exposure thresholds^[1,2]^. Adding to the complexity, water pipe smoking is often accompanied by co-exposures to other risk factors such as cigarette smoking, poor diet, and sedentary lifestyle, which may also contribute to cardiovascular dysfunction. Differentiating the specific impact of water pipe smoking on heart failure from these coexisting factors can be challenging. Moreover, the latency period between the initiation of water pipe smoking and the manifestation of heart failure symptoms may span years, further obscuring the causal relationship^[3]^. Another critical issue is the overlap in clinical presentation between water pipe smoking-induced heart failure and other forms of cardiac dysfunction. Symptoms such as fatigue, dyspnea, and fluid retention are nonspecific and can result from various cardiovascular or pulmonary conditions.

Traditional diagnostic tools, including echocardiography and electrocardiography, can identify ventricular dysfunction but do not differentiate the etiology. Additionally, standard laboratory tests such as B-type natriuretic peptide (BNP) and N-terminal pro-BNP (NT-proBNP) levels are elevated in heart failure regardless of the underlying cause, offering limited specificity for water pipe-related damage^[4]^. The lack of awareness among healthcare providers about the cardiovascular implications of water pipe smoking exacerbates diagnostic delays. Many clinicians are less familiar with the toxicological profile of water pipe smoke compared to cigarette smoke, leading to missed opportunities for early intervention. Educational efforts are needed to bridge this knowledge gap and encourage the inclusion of water pipe smoking history as a routine component of cardiovascular risk assessments^[5]^.

To address these diagnostic challenges, there is growing interest in identifying specific biomarkers that can detect water pipe smoking-induced heart damage at an early stage. Biomarkers are measurable biological indicators that provide insights into the underlying pathophysiology of a disease. In the context of water pipe smoking and heart failure, potential biomarkers fall into three broad categories: oxidative stress markers, inflammatory mediators, and myocardial injury indicators.

Oxidative stress plays a central role in the cardiovascular toxicity of water pipe smoke. The inhalation of water pipe smoke generates reactive oxygen species (ROS), leading to oxidative damage to myocardial cells and vascular endothelium. Biomarkers such as malondialdehyde (MDA), 8-isoprostane, and oxidized low-density lipoprotein (ox-LDL) have been investigated for their ability to reflect oxidative damage. Elevated levels of these biomarkers have been observed in both animal models and human studies of smoking-related cardiovascular diseases, suggesting their potential utility in detecting early myocardial injury in water pipe smokers^[6,7]^.

Inflammatory pathways are also significantly activated by water pipe smoke exposure. Chronic inflammation contributes to myocardial remodeling, fibrosis, and eventual heart failure. Key inflammatory biomarkers include C-reactive protein (CRP), interleukin-6 (IL-6), tumor necrosis factor-alpha (TNF-α), and matrix metalloproteinases (MMPs). Elevated CRP levels have been strongly associated with an increased risk of heart failure, while IL-6 and TNF-α have been implicated in the progression of ventricular dysfunction. Measuring these biomarkers could help identify water pipe smokers at high risk of developing heart failure^[8,10]^.

Myocardial injury can also be assessed through the measurement of cardiac-specific biomarkers. Troponins, particularly high-sensitivity cardiac troponin T (hs-cTnT), are well-established markers of myocardial injury and have been used extensively in diagnosing acute coronary syndromes. Recent studies suggest that low-level elevations in hs-cTnT may indicate subclinical myocardial damage in chronic smokers, including water pipe users. Similarly, heart-type fatty acid-binding protein (H-FABP) and soluble ST2 (sST2), both emerging biomarkers of myocardial stress and fibrosis, could provide additional diagnostic insights^[9,11]^.

In addition to traditional biomarkers, trimethylamine N-oxide (TMAO) is increasingly recognized as a key metabolic biomarker associated with cardiovascular disease and heart failure. TMAO is generated through hepatic oxidation of trimethylamine, a gut microbiota-derived metabolite from dietary precursors such as choline and carnitine^[6]^. Recent evidence suggests that smoking, including water pipe use, can influence gut microbial composition and systemic inflammation, potentially resulting in elevated circulating TMAO levels. High TMAO concentrations have been linked to endothelial dysfunction, enhanced platelet aggregation, and myocardial fibrosis—all of which contribute to heart failure pathophysiology^[12]^. Understanding the relationship between smoking and TMAO may offer additional diagnostic value and help elucidate one of the mechanistic pathways linking water pipe use to cardiovascular risk.

Despite their potential, the clinical implementation of these biomarkers faces several challenges. The specificity of oxidative stress and inflammatory markers for water pipe smoking-induced heart failure is limited, as these biomarkers are also elevated in other forms of cardiovascular and systemic diseases. Moreover, the cost and accessibility of advanced biomarker assays may hinder their routine use in resource-limited settings where water pipe smoking is prevalent. Standardization of assay techniques and establishment of reference ranges are necessary to improve these biomarkers’ reliability and clinical applicability^[12,13]^.

Emerging technologies, such as metabolomics and proteomics, offer new opportunities to identify novel biomarkers and elucidate the molecular pathways linking water pipe smoking to heart failure. Metabolomic profiling has revealed alterations in lipid metabolism, amino acid pathways, and oxidative stress markers in smokers, providing a broader understanding of the systemic impact of smoke exposure. Proteomic studies have similarly identified changes in protein expression related to inflammation, endothelial dysfunction, and myocardial injury. These approaches aim to uncover more specific and sensitive biomarkers tailored to water pipe smokers^[13,14]^.

## Therapeutic strategies and management

The management of heart failure in water pipe smokers aligns with general heart failure guidelines (see Table 4), including pharmacological and non-pharmacological strategies. Pharmacological treatments commonly include angiotensin-converting enzyme inhibitors (ACEIs) or angiotensin receptor blockers (ARBs) to reduce afterload and preload, beta-blockers to improve ventricular function and reduce sympathetic activation, and mineralocorticoid receptor antagonists (MRAs) to mitigate fluid retention and improve cardiac remodeling. Loop diuretics are often prescribed to manage symptoms of congestion and fluid overload^[1,2]^. These therapies are critical for stabilizing patients and improving long-term outcomes.

**Table 4:** Guidelines for Managing Heart Failure

Guideline Name	Category	Intervention	Dosage	Patient type	Indication	Class of recommendation	Level of evidence	Mechanism of action	Monitoring requirements	Comments/notes
ACC/AHA 2022	Lifestyle Changes	Sodium restriction	<2 g/day sodium	All patients	Reduce fluid retention	I	B	Decreases sodium overload	Monitor weight and fluid status	Emphasize on education and adherence
ESC 2021		Physical activity	Moderate-intensity exercise, tailored	Stable patients	Improve functional capacity	I	B	Enhances cardiovascular fitness	Evaluate tolerance and symptoms	Avoid in acute decompensation
ACC/AHA 2022	Pharmacological	ACE inhibitors	E.g., Enalapril 5-20 mg/day	HFrEF patients	Symptom relief and mortality reduction	I	A	Reduces afterload and preload	Monitor potassium and renal function	First-line therapy in HFrEF
ESC 2021		Beta-blockers	E.g., Bisoprolol 1.25-10 mg/day	HFrEF patients	Reduce mortality and hospitalizations	I	A	Reduces sympathetic overactivity	Monitor heart rate and BP	Start low and titrate slowly
ACC/AHA 2022		Diuretics	E.g., Furosemide 20-80 mg/day as needed	Patients with fluid overload	Symptom relief of congestion	I	B	Promotes fluid excretion	Monitor electrolytes and weight	Use lowest effective dose
ESC 2021	Device Therapy	CRT	Per manufacturer’s settings	HFrEF with LBBB	Improve symptoms and reduce mortality	I	A	Synchronizes ventricular contraction	Monitor device function	Requires specialized centers
ACC/AHA 2022		ICD	Per manufacturer’s settings	HFrEF and high-risk patients	Prevent sudden cardiac death	I	A	Prevents fatal arrhythmias	Regular device follow-up	For primary or secondary prevention
ESC 2021	Advanced Therapy	LVAD	Per manufacturer’s protocol	End-stage HF	Bridge to transplant or destination	IIa	B	Supports cardiac output	Monitor for infections and device complications	Limited to specialized centers
ACC/AHA 2022		Heart transplant	N/A	End-stage HF	Refractory to all other treatments	I	B	Restores normal cardiac function	Lifelong immunosuppressant therapy	Requires strict patient selection

Key: ACC/AHA: American College of Cardiology/American Heart Association; ESC: European Society of Cardiology; HFrEF: Heart Failure with Reduced Ejection Fraction; LBBB: Left Bundle Branch Block; CRT: Cardiac Resynchronization Therapy; ICD: Implantable Cardioverter Defibrillator; LVAD: Left Ventricular Assist Device. Source: Authors’ Creations.

However, specific considerations must be given to water pipe smokers due to the direct cardiovascular effects of the toxins they are exposed to. Carbon monoxide and other constituents of water pipe smoke exacerbate myocardial stress and hypoxia, requiring tailored management strategies. One emerging area of focus is the potential use of antioxidant therapies to counteract the oxidative stress induced by water pipe smoking. Reactive oxygen species (ROS) generated by water pipe smoke contribute significantly to myocardial damage and dysfunction. Antioxidant agents such as N-acetylcysteine, coenzyme Q10, and vitamins C and E have shown promise in reducing oxidative stress in preclinical and clinical studies. These antioxidants scavenge free radicals, prevent lipid peroxidation, and reduce oxidative damage to cardiac tissues^[3,4]^.

While further research is needed to establish their efficacy in the context of water pipe smoking, initial findings suggest that antioxidant therapies may complement standard heart failure treatment by addressing a critical underlying mechanism of disease progression. In addition to oxidative stress, inflammation plays a pivotal role in the pathophysiology of heart failure in water pipe smokers. Chronic exposure to water pipe smoke triggers the release of pro-inflammatory cytokines such as tumor necrosis factor-alpha (TNF-α), interleukin-6 (IL-6), and C-reactive protein (CRP). These inflammatory mediators contribute to endothelial dysfunction, myocardial fibrosis, and ventricular remodeling, exacerbating heart failure symptoms and progression^[5]^. Anti-inflammatory therapies offer a promising avenue for intervention.

Recent studies have explored the use of biologic agents targeting specific inflammatory pathways. For instance, TNF-α inhibitors such as etanercept and infliximab, initially developed for autoimmune diseases, have demonstrated potential in reducing systemic inflammation and improving cardiac function in certain subsets of heart failure patients^[6,7]^. Another class of anti-inflammatory agents under investigation is colchicine, a microtubule inhibitor traditionally used for gout. Colchicine has been shown to reduce cardiovascular inflammation by inhibiting neutrophil activation and IL-1β production. Clinical trials such as the COLCOT trial have provided evidence supporting the use of colchicine in reducing cardiovascular events in high-risk populations^[8]^.

Its applicability to water pipe smokers with heart failure remains an active research area. Still, its ability to target inflammation at multiple levels makes it an attractive candidate for further investigation. The potential of statins in managing heart failure associated with waterpipe smoking also warrants attention. In addition to their lipid-lowering effects, statins possess pleiotropic properties, including anti-inflammatory and antioxidant actions. These effects help mitigate the cardiovascular damage from water pipe smoke exposure. Studies have shown that statins reduce CRP levels, improve endothelial function, and decrease oxidative stress, making them a valuable adjunct to heart failure therapy in this population^[9,10]^.

Non-pharmacological interventions are equally important in managing heart failure in water pipe smokers. Lifestyle modifications, including smoking cessation, dietary changes, and regular physical activity, comprise comprehensive care’s cornerstone. Smoking cessation programs tailored to water pipe users, incorporating behavioral counseling and pharmacological aids such as nicotine replacement therapy or varenicline, are critical for reducing ongoing exposure to harmful toxins^[11]^. Effective cessation strategies can halt further cardiovascular damage and allow for better management of existing heart failure symptoms.

Cardiac rehabilitation programs offer additional support, combining supervised exercise training with education and psychological counseling. These programs have been shown to improve functional capacity, reduce hospital readmissions, and enhance the quality of life in heart failure patients. Tailoring these programs to address the unique needs of water pipe smokers, including addressing misconceptions about the safety of water pipe smoking, can further enhance their effectiveness^[12,13]^.

Emerging technologies, such as wearable devices for continuous monitoring of cardiac function, hold promise for improving the management of heart failure in this population. These devices can help detect early signs of decompensation, allowing for timely intervention and reducing the risk of hospitalization. Integration of telemedicine into heart failure care can also improve access to specialist care, particularly for patients in low-resource settings where water pipe smoking is prevalent^[14]^.

Future therapeutic strategies may benefit from a personalized medicine approach, considering genetic and epigenetic factors influencing individual susceptibility to heart failure and response to treatment. Advances in genomics and transcriptomics could pave the way for targeted therapies that address the specific molecular pathways activated by water pipe smoke exposure. For instance, identifying genetic variants associated with increased oxidative stress or inflammation could guide the use of specific antioxidants or anti-inflammatory agents in high-risk individuals^[15]^.

## Behavioral and socio-cultural factors

Cultural acceptance plays a significant role in the proliferation of water pipe smoking. In Middle Eastern and South Asian cultures, water pipe smoking is often intertwined with social gatherings, religious celebrations, and traditional customs. It is not uncommon for families and friends to gather in cafes, homes, or cultural events where water pipe smoking is a focal point. This cultural normalization extends beyond traditional societies and into global diaspora communities, where water pipe smoking maintains cultural identity and heritage. Such acceptance fosters an environment where the health risks associated with waterpipe smoking are either underestimated or overlooked entirely. The communal aspect, often seen as a symbol of hospitality and togetherness, further normalizes its use, even among individuals who might otherwise avoid tobacco products^[1,2]^.

The social drivers of water pipe smoking are equally compelling. Water pipe smoking has become a trendy social activity in contemporary contexts, particularly among youth and young adults. Hookah lounges and cafes have proliferated in urban areas worldwide, creating spaces where individuals can gather and enjoy flavored tobacco in a relaxed environment. The perception of water pipe smoking as a more socially acceptable and less harmful alternative to cigarettes has been reinforced by aggressive marketing strategies and the availability of appealing flavors, such as fruit, mint, and chocolate. These factors make water pipe smoking particularly attractive to younger demographics, including those who might not have otherwise initiated tobacco use. Peer influence and the desire to participate in social bonding experiences further drive adoption rates, creating a cycle of normalization and dependency^[3,4]^.

Behavioral factors, including misperceptions about the health risks of water pipe smoking, also contribute to its widespread use. Many individuals believe that the water used in the hookah apparatus filters out harmful toxins, making it a safer option than cigarettes. This belief is scientifically inaccurate and perpetuates a dangerous underestimation of the associated health risks. Research indicates that a single water pipe session can expose users to significantly higher levels of carbon monoxide, tar, and other harmful substances compared to smoking a cigarette^[5,6]^. Despite this, the sensory appeal of water pipe smoking—its smooth inhalation, aromatic flavors, and visually engaging setup—contributes to its behavioral attractiveness and reinforces its perceived harmlessness.

These behavioral and socio-cultural drivers have significant implications for public health intervention strategies. Efforts to reduce the prevalence of water pipe smoking must address the deeply rooted cultural and social norms that perpetuate its use. Traditional tobacco control measures, such as warning labels, taxation, and advertising restrictions, have proven effective for cigarette smoking but are often less impactful for water pipes due to their unique cultural and social context. For example, warning labels on tobacco packaging may not effectively reach users who frequent hookah lounges, where tobacco is often served in bulk without clear labeling. Similarly, taxation policies targeting cigarettes may not sufficiently encompass the water pipe tobacco market, which is often sold and consumed in unregulated environments^[7,8]^.

Public health campaigns aimed at reducing water pipe smoking must be tailored to the specific socio-cultural context of target populations. In regions where water pipe smoking is culturally ingrained, interventions should focus on education and awareness initiatives that highlight the health risks while respecting cultural sensitivities. Engaging community leaders, religious figures, and other influential individuals can help shift perceptions and encourage healthier behaviors. For example, campaigns in Middle Eastern communities could collaborate with religious leaders to emphasize the health risks of waterpipe smoking in the context of broader moral and ethical teachings. Similarly, working with youth organizations and schools can help counter the social appeal of water pipe smoking among younger populations^[9,10]^.

Another critical factor is the role of media and technology in shaping perceptions of water pipe smoking. Social media platforms, for instance, have become significant channels for the promotion and glamorization of hookah use. Posts featuring aesthetically pleasing setups, exotic flavors, and group activities create an aspirational image that appeals to younger audiences. Counteracting this trend requires innovative strategies that leverage the same platforms to disseminate evidence-based information about the risks of water pipe smoking. For instance, using influencers and digital campaigns to highlight the dangers of water pipe smoking and share testimonials from affected individuals can effectively reach a broader audience^[11,12]^.

Compliance with public health initiatives also depends on addressing the regulatory challenges associated with waterpipe smoking. Unlike cigarettes, which are often consumed individually, water pipe smoking is a communal activity that complicates enforcement of tobacco control measures. For example, smoke-free policies may be difficult to implement in hookah lounges and private gatherings, where the activity is deeply embedded in social practices. Ensuring compliance requires robust legislation that includes water pipe smoking within the scope of existing tobacco control laws and establishes clear guidelines for enforcement. This could involve licensing requirements for hookah lounges, restrictions on indoor smoking, and penalties for non-compliance^[13,14]^.

Moreover, interventions must consider the economic drivers of water pipe smoking, particularly in low- and middle-income countries where it is often a source of livelihood for small business owners. Hookah lounges and tobacco vendors represent a significant economic sector in some regions, creating resistance to regulatory measures that could threaten their income. Public health strategies should include measures to support alternative livelihoods and provide economic incentives for compliance with regulations. For instance, offering subsidies or grants to businesses transitioning from selling water pipe tobacco could help mitigate resistance and foster a more supportive environment for tobacco control initiatives^[15,16]^.

Despite these efforts, significant research gaps remain regarding the behavioral and socio-cultural drivers of water pipe smoking. Most studies to date have focused on cigarette smoking, leaving a critical need for more targeted research on waterpipe use. Understanding the specific motivations, cultural contexts, and barriers to cessation can help inform more effective interventions. Additionally, longitudinal studies are needed to assess the long-term health impacts of water pipe smoking and evaluate the effectiveness of existing public health initiatives^[17,18]^.

## Role of healthcare professionals

Healthcare providers must base their interventions on robust, evidence-based practices when addressing the cardiovascular risks of water pipe smoking. Studies indicate that water pipe users are exposed to significantly higher toxicants compared to cigarette smokers—1.7 times more nicotine and 9 times more carbon monoxide per session—which correlates with increased risks of ventricular dysfunction, endothelial damage, and systemic inflammation^[24-26]^. These pathophysiological changes are well-established precursors of heart failure and other cardiovascular events.

To address this, clinicians should routinely screen for water pipe use using validated tobacco use questionnaires during patient evaluations. This ensures comprehensive risk stratification and facilitates early identification of at-risk individuals. Data show that incorporating water pipe screening into routine history-taking increases detection rates by over 30% in populations under 40 years^[27,28]^.

Once identified, healthcare providers should employ structured, evidence-based cessation strategies. When adapted to account for cultural nuances, motivational interviewing has improved tobacco cessation rates by up to 20%^[29]^. In addition, brief interventions lasting less than 10 minutes have demonstrated a statistically significant impact on reducing water pipe use, particularly when reinforced over follow-up visits^[30]^.

Clinicians should also prioritize targeted education using clear, quantifiable data. For example, highlighting that a single water pipe session delivers smoke volume equivalent to 100–200 cigarettes can significantly enhance users’ risk perception^[31-33]^.

Periodic cardiovascular assessments, including echocardiograms and serum biomarker testing (e.g., CRP, troponins), can facilitate early detection of subclinical disease in habitual users. Evidence supports the integration of such screening into annual check-ups for known water pipe users, as it improves early intervention rates by over 25%^[34-36]^.

Finally, providers should actively contribute to public health surveillance and community-level interventions. This includes participating in policy discussions and education campaigns where empirical data is used to counter misconceptions. Consistent messaging backed by data improves public awareness and shifts behavior at a population level.

## Future research directions

While the clinical and epidemiological evidence linking water pipe smoking to heart failure is compelling, several gaps in knowledge remain. One notable limitation is the over-reliance on observational studies. Although valuable for hypothesis generation, these studies are prone to confounding and bias. For example, many fail to adequately account for co-exposures, such as concurrent use of other tobacco products or environmental pollutants, which may obscure the observed associations. This lack of thorough control over confounding factors undermines the strength of causal inferences. Another issue is the heterogeneity in study designs and methodologies. Variations in defining water pipe smoking, including frequency, duration, and intensity, create challenges for cross-study comparisons.

Similarly, inconsistencies in how cardiovascular outcomes, such as heart failure phenotypes, are measured or categorized hinder the synthesis of findings. Standardized definitions and methodological frameworks are essential to improve the comparability and reliability of future studies. The majority of current research focuses on the acute or short-term effects of water pipe smoking, leaving the long-term cardiovascular consequences underexplored.

To address this gap, future research should prioritize longitudinal cohort studies that monitor water pipe smokers over extended periods. These studies are critical for establishing causal relationships and evaluating the progression of cardiovascular abnormalities such as ventricular dysfunction, chronic inflammation, and oxidative stress. Ongoing or planned studies incorporating repeated biomarker assessments and advanced cardiac imaging will be particularly valuable in identifying at-risk populations and assessing the effectiveness of early interventions.

These studies could clarify the temporal relationship between waterpipe smoking and heart failure development and identify potential windows for intervention or reversal of damage. Geographical generalizability is another concern. Much of the available evidence is derived from the Middle East and North Africa (MENA) region, where water pipe smoking is deeply embedded in cultural norms and often practiced daily.

However, the health impacts in Western populations, where the habit is more recreational and sporadic, may differ significantly. Expanding research efforts to diverse populations would provide a more comprehensive understanding of the global burden of water pipe smoking. Additionally, future investigations should include non-English language studies and foster collaborations with researchers from non-English-speaking countries to minimize language bias and enhance global data representation.

Mechanistic studies exploring the specific pathways by which water pipe smoking contributes to heart failure are also lacking. While oxidative stress and inflammation have been implicated, the relative contributions of these pathways to distinct heart failure phenotype—such as heart failure with preserved ejection fraction (HFpEF) versus reduced ejection fraction (HFrEF)—remain unclear. Experimental research using animal models and advanced imaging techniques could provide valuable insights into these mechanisms and help tailor preventive or therapeutic strategies.

Finally, addressing public health misconceptions surrounding water pipe smoking is critical. Many individuals perceive water pipe smoking as a safer alternative to cigarettes, a belief often perpetuated by cultural narratives and aggressive marketing. This misconception undermines efforts to raise awareness about its significant cardiovascular risks. Effective public health campaigns must employ culturally sensitive communication strategies, leveraging clear scientific evidence to dispel myths and educate the public about the dangers of water pipe smoking.

## Concluding remarks

Water pipe smoking poses a significant risk to cardiovascular health by contributing to ventricular dysfunction, oxidative stress, and systemic inflammation—key mechanisms implicated in heart failure. Current evidence underscores the urgent need for targeted public health interventions, stricter regulations, and further research to mitigate its growing impact on the global cardiovascular disease burden.

## Call to action

Urgent action is needed to address the underestimated cardiovascular risks of water pipe smoking. Researchers, clinicians, and policymakers must prioritize awareness, stricter regulations, and targeted interventions to mitigate its impact on heart health.

## Data Availability

This published article and its supplementary information files include all data generated or analyzed during this study.

## References

[1] Rezk-Hanna M Rossman MJ Ludwig K. Electronic hookah (waterpipe) vaping reduces vascular endothelial function: the role of nicotine. Am J Physiol Heart Circ Physiol 2024;326:H490–H496. 38133618 10.1152/ajpheart.00710.2023 PMC11219048

[2] Tsao CW Aday AW Almarzooq ZI. Heart disease and stroke statistics-2023 update: a report from the American heart association. Circulation 2023;147:e93–e621. [Erratum in Circulation 147: e622,2023, and in Circulation 148: e4, 2023]. 36695182 10.1161/CIR.0000000000001123 PMC12135016

[3] Mahfooz K Vasavada AM Joshi A. Waterpipe use and its cardiovascular effects: a systematic review and meta-analysis of case-control, cross-sectional, and non-randomized studies. Cureus 2023;15:e34802. 36915837 10.7759/cureus.34802 PMC10008028

[4] Jawad M Shihadeh A Nakkash RT. Philip morris patents ‘harm reduction’ electronic waterpipe. Tob Control 2021;30:473. 10.1136/tobaccocontrol-2020-055885 32587110

[5] Varghese J Muntode Gharde P. A comprehensive review on the impacts of smoking on the health of an individual. Cureus 2023;15:e46532. 37927763 10.7759/cureus.46532 PMC10625450

[6] Latif F Mubbashir A Khan MS. Trimethylamine N-oxide in cardiovascular disease: pathophysiology and the potential role of statins. Life Sci 2025;361:123304. 39672256 10.1016/j.lfs.2024.123304

[7] Evans WG. Anti-smoking campaigns. Sadj 2013;68:58–59. https://pubmed.ncbi.nlm.nih.gov/23951764/. 23951764

[8] Dube SR Pathak S Nyman AL. Electronic cigarette and electronic hookah: a pilot study comparing two vaping products. Prev Med Rep 2015;2:953–58. 26740911 10.1016/j.pmedr.2015.10.012 PMC4698898

[9] Bhatnagar A Maziak W Eissenberg T. Water pipe (hookah) smoking and cardiovascular disease risk: a scientific statement from the American heart association. Circulation 2019;139:e917–e936. 30845826 10.1161/CIR.0000000000000671 PMC6600812

[10] Rezk-Hanna M Toyama J Ikharo E. E-hookah versus e-cigarettes: findings from wave 2 of the PATH study (2014-2015). Am J Prev Med 2019;57:e163–e173. 31564602 10.1016/j.amepre.2019.05.007

[11] Fetterman JL Weisbrod RM Feng B. Flavorings in tobacco products induce endothelial cell dysfunction. Arterioscler Thromb Vasc Biol 2018;38:1607–15. 29903732 10.1161/ATVBAHA.118.311156 PMC6023725

[12] Bal I Balci N Sorguc C. Trimethylamine N-oxide (TMAO) and TNF-α levels in periodontal disease associated with smoking. Oral Dis 2025. 10.1111/odi.15262 39887517

[13] Sykes AP Brampton C Klee S. An investigation into the effect and mechanisms of action of nicotine in inflammatory bowel disease. Inflamm Res 2000;49:311–19. 10959551 10.1007/s000110050597

[14] Benowitz NL Burbank AD. Cardiovascular toxicity of nicotine: implications for electronic cigarette use. Trends Cardiovasc Med 2016;26:515–23. 27079891 10.1016/j.tcm.2016.03.001 PMC4958544

[15] Münzel T Hahad O Kuntic M. Effects of tobacco cigarettes, e-cigarettes, and waterpipe smoking on endothelial function and clinical outcomes. Eur Heart J 2020;41:4057–70. 32585699 10.1093/eurheartj/ehaa460 PMC7454514

[16] Rezk-Hanna M Seals DR Rossman MJ. Ascorbic acid prevents vascular endothelial dysfunction induced by electronic hookah (waterpipe) vaping. J Am Heart Assoc 2021;10:e019271. 33615833 10.1161/JAHA.120.019271 PMC8174254

[17] Shihadeh A Azar S Antonios C. Towards a topographical model of narghile water-pipe cafe smoking: a pilot study in a high socioeconomic status neighborhood of Beirut, Lebanon. Pharmacol Biochem Behav 2004;79:75–82. 15388286 10.1016/j.pbb.2004.06.005

[18] Antinozzi M Giffi M Sini N. Cigarette smoking and human gut microbiota in healthy adults: a systematic review. Biomedicines 2022;10:510. 35203720 10.3390/biomedicines10020510 PMC8962244

[19] Jain H Marsool MDM Goyal A. Unveiling the relationship between gut microbiota and heart failure: recent understandings and insights. Curr Probl Cardiol 2024;49:102179. 37923029 10.1016/j.cpcardiol.2023.102179

[20] Freeberg KA Ludwig KR Chonchol M. NAD(+)-boosting compounds enhance nitric oxide production and prevent oxidative stress in endothelial cells exposed to plasma from patients with COVID-19. Nitric Oxide 2023;140-141:1–7. 37657532 10.1016/j.niox.2023.08.003 PMC10840929

[21] Murray KO Ludwig KR Darvish S. Chronic mitochondria antioxidant treatment in older adults alters the circulating milieu to improve endothelial cell function and mitochondrial oxidative stress. Am J Physiol Heart Circ Physiol 2023;325:H187–H194. 37326998 10.1152/ajpheart.00270.2023 PMC10312314

[22] Neunteufl T Heher S Kostner K. Contribution of nicotine to acute endothelial dysfunction in long-term smokers. J Am Coll Cardiol 2002;39:251–56. 11788216 10.1016/s0735-1097(01)01732-6

[23] Chalon S Moreno HJ Benowitz NL. Nicotine impairs endothelium-dependent dilatation in human veins in vivo. Clin Pharmacol Ther 2000;67:391–97. 10801248 10.1067/mcp.2000.105153

[24] Guan ZZ Yu WF Nordberg A. Dual effects of nicotine on oxidative stress and neuroprotection in PC12 cells. Neurochem Int 2003;43:243–49. 12689604 10.1016/s0197-0186(03)00009-3

[25] Crowley-Weber CL Dvorakova K Crowley C. Nicotine increases oxidative stress, activates NF-kappaB and GRP78, induces apoptosis and sensitizes cells to genotoxic/xenobiotic stresses by a multiple stress inducer, deoxycholate: relevance to colon carcinogenesis. Chem Biol Interact 2003;145:53–66. 12606154 10.1016/s0009-2797(02)00162-x

[26] Benowitz NL. Nicotine addiction. N Engl J Med 2010;362:2295–303. 20554984 10.1056/NEJMra0809890 PMC2928221

[27] Benowitz NL Fraiman JB. Cardiovascular effects of electronic cigarettes. Nat Rev Cardiol 2017;14:447–56. 28332500 10.1038/nrcardio.2017.36 PMC5519136

[28] Chaumont M de Becker B Zaher W. Differential effects of e-cigarette on microvascular endothelial function, arterial stiffness and oxidative stress: a randomized crossover trial. Sci Rep 2018;8:10378. 29991814 10.1038/s41598-018-28723-0 PMC6039507

[29] Schulz H Harder V Ibald-Mulli A. Cardiovascular effects of fine and ultrafine particles. J Aerosol Med 2005;18:1–22. 15741770 10.1089/jam.2005.18.1

[30] McGraw KE Riggs DW Rai S. Exposure to volatile organic compounds - acrolein, 1,3-butadiene, and crotonaldehyde - is associated with vascular dysfunction. Environ Res 2021;196:110903. 33636185 10.1016/j.envres.2021.110903 PMC8119348

[31] George J Hussain M Vadiveloo T. Cardiovascular effects of switching from tobacco cigarettes to electronic cigarettes. J Am Coll Cardiol 2019;74:3112–20. 31740017 10.1016/j.jacc.2019.09.067 PMC6928567

[32] Ikonomidis I Vlastos D Kourea K. Electronic cigarette smoking increases arterial stiffness and oxidative stress to a lesser extent than a single conventional cigarette: an acute and chronic study. Circulation 2018;137:303–06. 29335291 10.1161/CIRCULATIONAHA.117.029153

[33] Langley TE McNeill A Lewis S. The impact of media campaigns on smoking cessation activity: a structural vector autoregression analysis. Addiction 2012;107:2043–50. 22632403 10.1111/j.1360-0443.2012.03958.x

[34] Peruga A Molina X Delgado I. Compliance with the smoking ban in enclosed, semiopen and open areas of workplaces and public places in Chile. Tob Control 2021;30:570–73. 32703800 10.1136/tobaccocontrol-2020-055632

[35] Fong GT Craig LV Guignard R. Evaluation of the smoking ban in public places in France one year and five years after its implementation: findings from the ITC France survey. Bull Epidemiol Hebd (Paris) 2013;20:217–23. https://pubmed.ncbi.nlm.nih.gov/24803715/. 24803715 PMC4009376

[36] Chen MY. The negative impact of parental smoking on adolescents’ health-promoting behaviors: a cross-sectional study. Int J Environ Res Public Health 2021;18:2514. 33802561 10.3390/ijerph18052514 PMC7967525

